# Data center TCP dataset

**DOI:** 10.1016/j.dib.2024.110522

**Published:** 2024-05-15

**Authors:** Jan Fesl, Tereza Čapková, Michal Konopa, Ladislav Beránek, Jan Fiala, Michal Houda, Petr Chládek, Jana Klicnarová, Radim Remeš, Marek Šulista, Klára Vocetková, Marie Feslová

**Affiliations:** a Faculty of Economics, Department of Applied Mathematics and Informatics, University of South Bohemia in České Budějovice, Czechia; b Faculty of Information Technology, Department of Computer Systems, Czech Technical University in Prague, Czechia

**Keywords:** TCP, Congestion control, Data center, Identification, Machine learning

## Abstract

In this paper, we would like to introduce a unique dataset that covers thousands of network flow measurements realized through TCP in a data center environment. The TCP protocol is widely used for reliable data transfers and has many different versions. The various versions of TCP are specific in how they deal with link congestion through the congestion control algorithm (CCA). Our dataset represents a unique, comprehensive comparison of the 17 currently used versions of TCP with different CCAs. Each TCP flow was measured precisely 50 times to eliminate the measurement instability. The comparison of the various TCP versions is based on the knowledge of 18 quantitative attributes representing the parameters of a TCP transmission. Our dataset is suitable for testing and comparing different versions of TCP, creating new CCAs based on machine learning models, or creating and testing machine learning models, allowing the identification and optimization of the currently existing versions of TCP.

Specifications Table

**Table:** 

Subject	Computer Networks and Communications Computer Science Machine Learning
Specific subject area	Data from a real network traffic based on 17 different versions of TCP measured as a consequence of a 20-dimensional vector.
Type of data	Textual (Compressed CSV files)
How the data were acquired	The data was acquired from real network BSD Sockets, which provided two-sided TCP communication. The data was parsed and retransformed in easily-readable CSV files.
Data format	RAW
Description of data collection	The data was measured for approximately three months within the real computer network consisting of active network devices, i.e., routers and switches connected to the optic communication lines. The average achieved network throughput was in units of megabytes per second.
Data source location	University of South Bohemia Faculty of Economics, Department of Applied Mathematics and Informatics Studentská 13, České Budějovice Czech Republic GPS: 48.978843867004386, 14.451743105975511
Data accessibility	Repository name: Zenodo Data identification number: 10.5281/zenodo.10894768 Direct URL to data: https://zenodo.org/records/10894768 The dataset is freely available for usage without limitation.

## 1 Value of the Data

• TCP version comparison and suitability for data center (DC) networks.
• The dataset [1] contains sufficient samples to train machine learning models designed to identify the used CAA.
• Data analysts or network engineer experts can use the dataset.
• The dataset can be a benchmark for comparing the quality of different CAA algorithms.
• The use of the dataset lies in the possibility of speeding up the research, as it took three months to acquire and many people to gather resources for the dataset assembly and verification.
• The dataset is free of malicious content and is freely distributable*.*

## 2 Background

The main reason and motivation for creating this dataset was the possibility of comparing different versions of TCP in the sense of considering their usability in the environment of real data centers (DCs).

Currently, there exist many versions of TCP based on various principles.

Examples of traditional round trip time (RTT) based TCPs are NewReno [2], Cubic [3], BBR [4], Vegas [5], Low-Priority [6], and New Vegas [7]).

Data Center TCP (DTCP) [8] and SDTCP [9] are the first examples of TCP variants proposed especially for data centers (DCs). Another example of a TCP research initiative is the OpenTCP variant [10] proposed explicitly for SDN-based DC networks.

TCP versions (such as Hybla [11], Illinois [12], BIC [13], NewReno [14], Westwood+ [15], Veno [16], HighSpeed [17], Scalable [18], YeAH [19], and HTCP [20]) use various criteria (achievable throughput, RTT, fairness, friendliness, or compatibility with other protocols).

Based on our best knowledge, no similar data set exists that provides similar information freely available for download.

## 3 Data Description

**Fig. 1:**
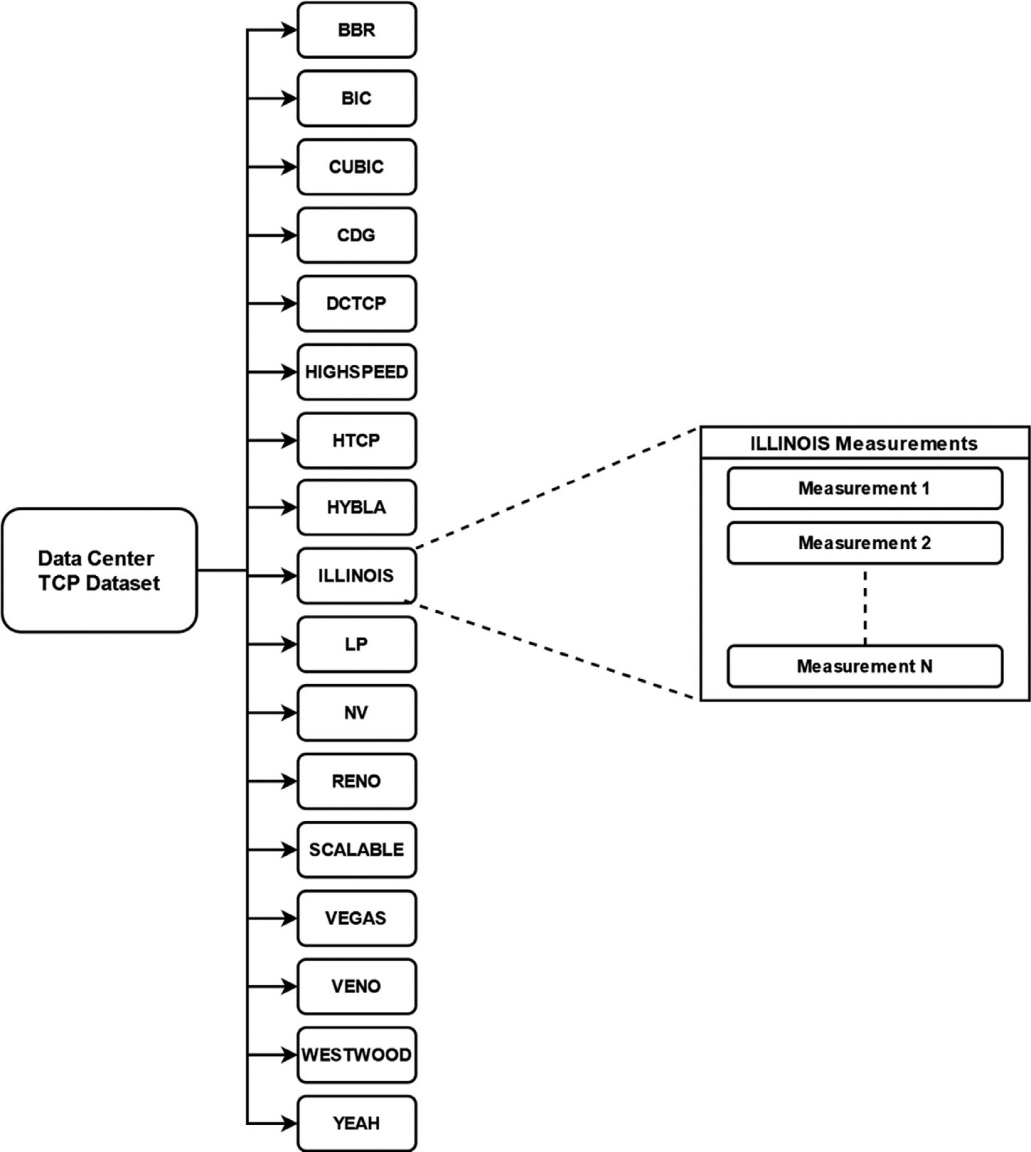
Data Center TCP Dataset structure. The dataset contains data of measurements from 17 different versions of TCP.

The basic structure of our dataset is shown in Fig. 1. The dataset is divided into a total of 17 different subdirectories that represent measurements of one TCP protocol variant. All measurements were performed under the same conditions, with line capacity reserved only for that measurement. The link capacity was deliberately limited to 16 Mbps to make it easy to achieve link congestion or to skew the corresponding CAA. All measurements were repeated 50 times to eliminate measurement errors. Each measurement is stored in a single CSV file.

Each CSV file contains a specific TCP flow measurement with a duration of approx. 15 min of data records. A vector of 18 parameters represents each record of measurement. The meaning and explanation of all parameters are presented in Table 1.

**Table 1:** The vector of classification parameters is preprocessed for each TCP stream in the dataset.

Num	Name	Meaning
1	wscale	The TCP window scale option is an option to increase the receive window size allowed in TCPl above its former maximum value of 65,535 bytes.
2	rto	Retransmission time out (rto) is the time set for acknowledgment of a particular packet to be received; after this time, the packet is declared lost and retransmitted. This value is in milliseconds.
3	rtt	Round-trip time (rtt), also known as the round-trip delay time, is defined as a metric that measures the amount of time it takes for a data packet to be sent plus the amount of time it takes for an acknowledgment of that packet to be received. The value represents the average rtt in milliseconds.
4	pmtu	Path maximum transfer unit (pmtu) is the maximum transmission unit (mtu) size on the network path between two Internet Protocol (IP) hosts, usually with the goal of avoiding IP fragmentation.
5	rmss	The receiver maximum segment size (rmss) is the size of the largest segment the receiver is willing to accept. This is the value specified in the MSS option sent by the receiver during connection startup.
6	advmss	The number of bytes advertised in the MSS option to the receiver during connection establishment and, therefore, the maximum segment size that will be sent from the sender.
7	cwnd	Congestion Window (cwnd) is the amount of data the TCP can send before receiving an acknowledgment packet (ACK).
8	ssthresh	The slow start threshold (ssthresh) value determines the (de)activation of TCP slow start.
9	bytes_sent	The number of bytes sent from the sender to the receiver. Starts from 0. This value is cumulative.
10	bytes_retrans	The number of resent bytes from the sender to the receiver. This value is cumulative.
11	bytes_acked	The number of received bytes confirmed by the receiver to the server. This value is cumulative.
12	segs_out	The number of TCP segments sent from the sender to the receiver. This value is cumulative.
13	segs_in	The number of TCP segments received by the sender from the receiver. This value is cumulative.
14	data_segs_out	The number of sent segments from the sender to the receiver containing a positive length data segment. This value is cumulative.
15	lastrcv	How much time since the TCP flow start has elapsed in milliseconds.
16	delivered	The count of TCP segments sent and confirmed by the receiver. This value is cumulative.
17	rcv_space	Value in bytes used in TCPʼs internal auto-tuning to grow socket buffers based on how much data the kernel estimates the sender can send.
18	rcv_ssthresh	The size of sshthresh at the receiver side.

In total, the data set contains 8350 different measurements for 17 different TCP versions. The total time of pure measurement was approximately more than 5 days.

**Fig. 2:**
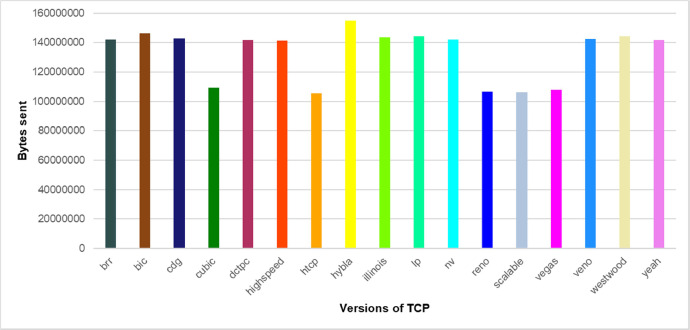
The comparison of the total amount of sent bytes by various versions of TCP during 15 min (each column represents a specific TCP version). Some TCP versions substantially exceed the others.

**Fig. 3:**
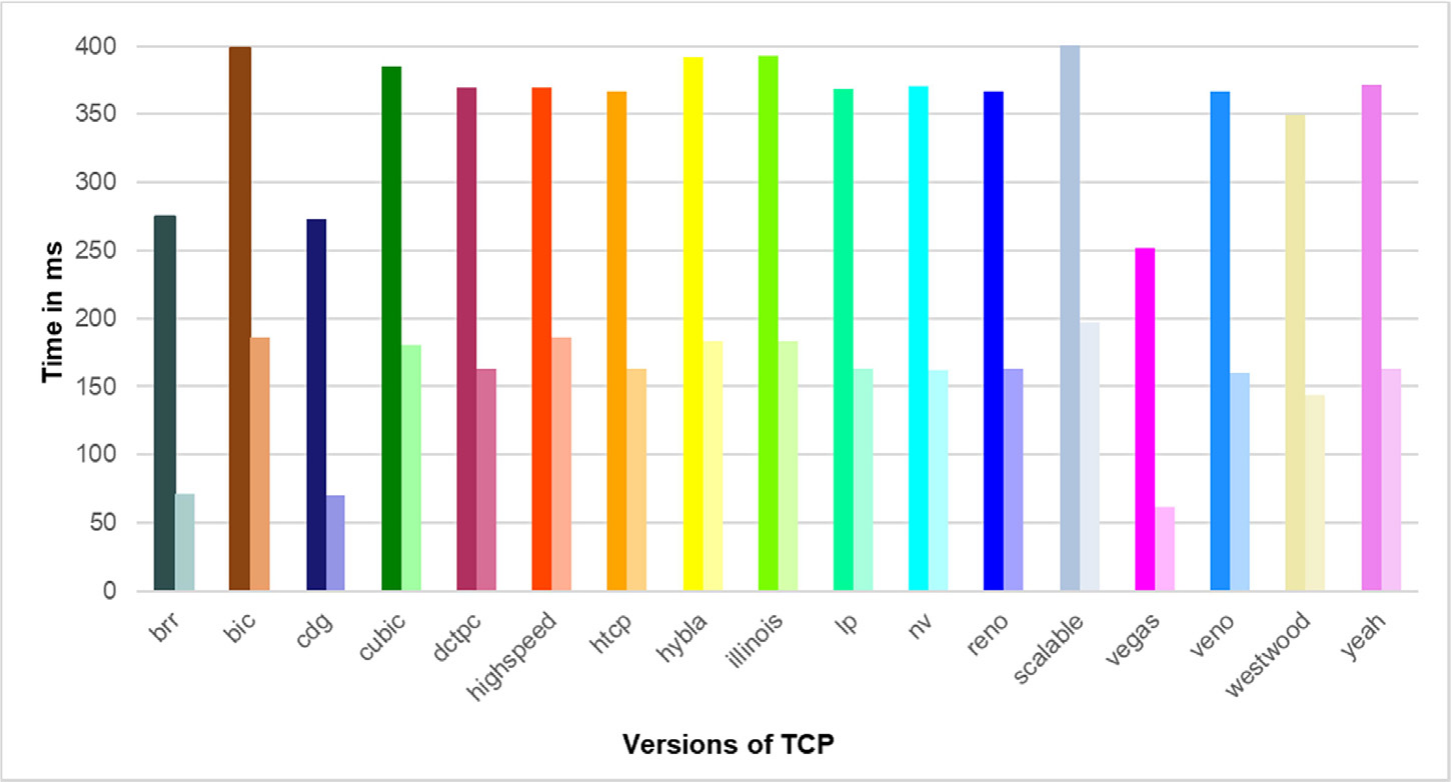
The comparison of rto/rtt values. Each couple of columns represents the rto/rtt values achieved for a specific TCP version. Rto/rtt values fundamentally impact the correct settings of the cwnd value.

**Fig. 4:**
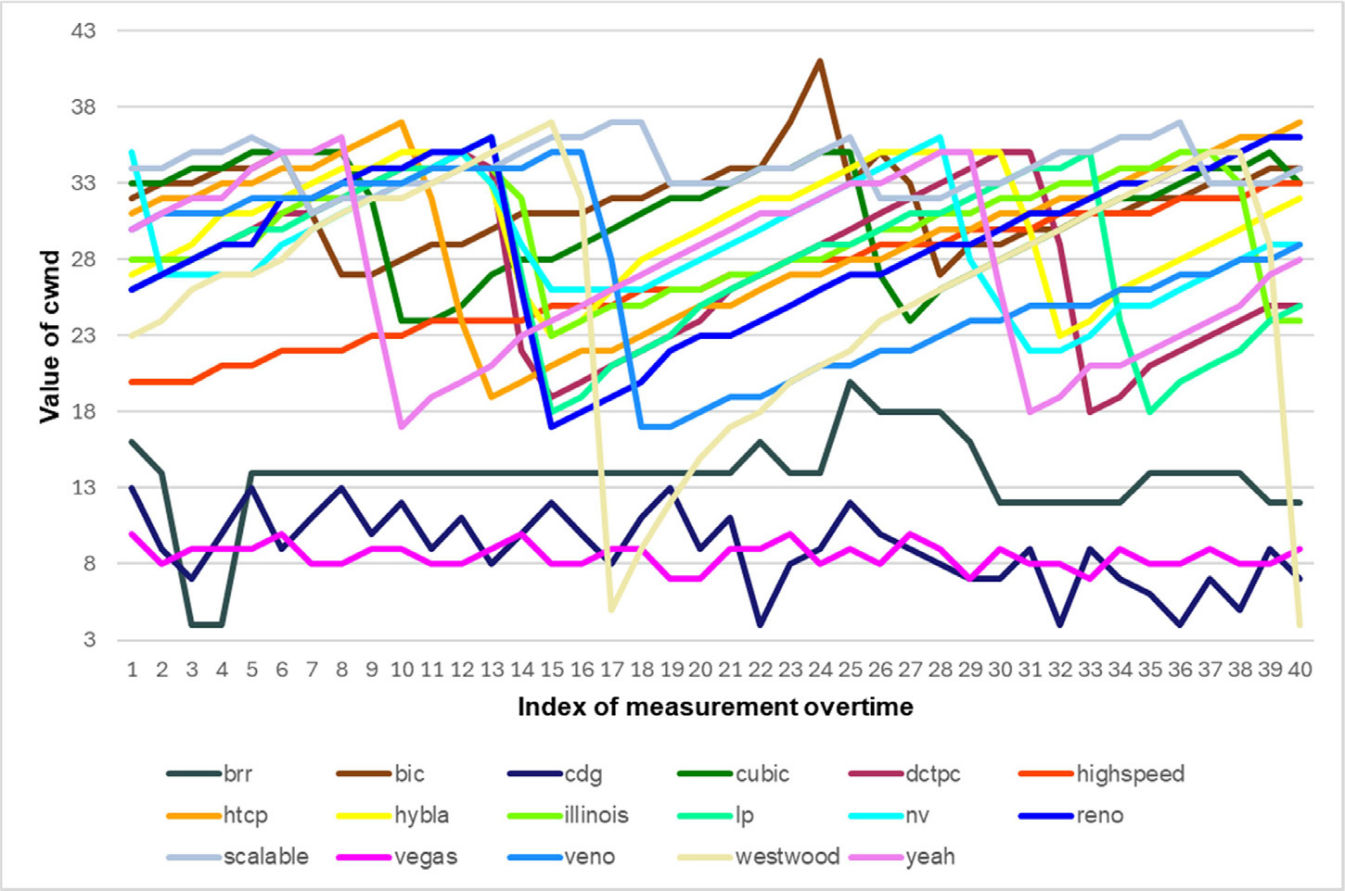
The comparison of cwnd values for various versions of TCP. Cwnd value directly affects the communication line congestion.

The graphs below show a partial information overview that can be retrieved from our dataset. In Fig. 2, there is an overall comparison of sent bytes by various versions of TCP. Our measurements show TCP Hybla can send the maximum amount of data. Fig. 3 shows the different combinations of values for rtt and rto during the measurement runtime. Rtt resp. rto values are important for setting the cwnd value computed by a specific CAA. Finally, Fig. 4 depicts a comparison of different cwnd value changes for different TCP versions during the measurement. The correct cwnd value is important for communication line congestion avoidance.

## 4 Experimental Design, Materials, and Methods

**Fig. 5:**
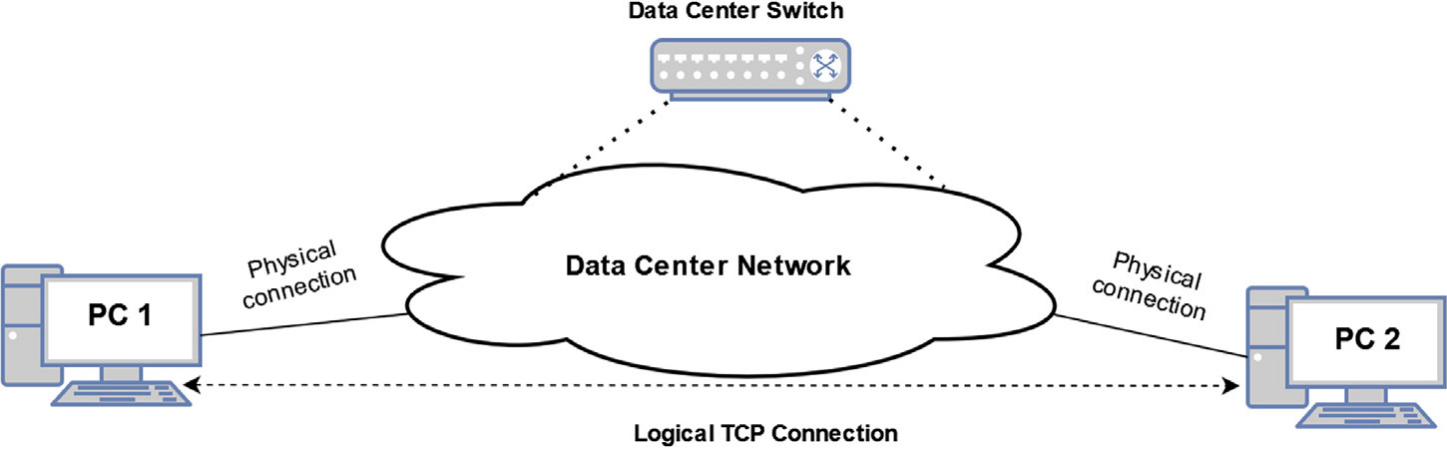
TCP flow measurement scheme. The line capacity limitation achieved the line congestion.

This section describes how we gathered the data and used the environment. The DC environment topology is depicted in Fig. 5. A physical connection between two desktops and the DC network was established. The line capacity between PC1 and PC2 was limited to 16 Mbps at Data Center Switch due to the easy establishment of the line congestion. The line capacity between PC1 and PC2 was isolated and guaranteed for the entire measurement time.

The measurements were technically performed in the following scenario. PC1 and PC2 contained the installed operating system Debian 11 with an installed package of the Iperf 3 tool and socket statistics (ss) tool. The Iperf 3 tool is currently considered a standard tool for network throughput measurement. The tool allows us to set many parameters, like the transport protocol (TCP or UDP), the number of parallel connections, maximum measurement time, etc. The ss tool was used during the measurement time to get values for parameters related to the specific TCP connection. The data retrieved by ss was periodically stored in a log file. The meaning of all TCP connection parameters it is possible to find in Table 1.

All measurements were performed separately in batches for each TCP version. Before the measurement started, the required TCP version was specified as the Linux kernel parameter in the /proc/sys/net/ipv4/tcp_congestion_control file. In this case, the kernel version 5.10.1 was used.

Each TCP batch measurement was performed in this way:

1. Immediately before the execution of the Iperf 3 tool, an executed ss tool started storing the TCP connection statistics into a textual log file. The Iperf 3 tool always created just only one logical TCP connection.
2. The step 1 was repeated 50 times.
3. CSV files suitable for the subsequent processing were generated from all log files. All CSV files were inserted into the data set.

## Limitations

None.

## Ethics Statement

Our work does not involve studies with animals and humans.

## CRediT authorship contribution statement

**Jan Fesl:** Data curation, Conceptualization, Methodology, Writing – original draft, Supervision. **Tereza Čapková:** Visualization. **Michal Konopa:** Software. **Ladislav Beránek:** Methodology. **Jan Fiala:** Data curation. **Michal Houda:** Data curation. **Petr Chládek:** Data curation. **Jana Klicnarová:** Methodology, Supervision. **Radim Remeš:** Software. **Marek Šulista:** Data curation. **Klára Vocetková:** Data curation. **Marie Feslová:** Visualization, Data curation.

## Data Availability

Data Center TCP Dataset (Original data) (Zenodo).

## Appendix Supplementary materials

Image, application 1

Image, application 2
